# Delayed chylopericardium after radical surgery for esophageal cancer: a case report

**DOI:** 10.3389/fonc.2023.1163618

**Published:** 2023-07-12

**Authors:** Pengjie Yang, Rui Han, Benben Zhu, Yu Wu, Bater Han

**Affiliations:** ^1^ Department of Thoracic Surgery, Peking University Cancer Hospital Inner Mongolia Hospital/Cancer Hospital Affiliated to Inner Mongolia Medical University, Hohhot, China; ^2^ Department of Thoracic Surgery, National Cancer Center/National Clinical Research Center for Cancer/Cancer Hospital, Chinese Academy of Medical Sciences and Peking Union Medical College, Beijing, China; ^3^ Department of Pharmacy, Peking University Cancer Hospital Inner Mongolia Hospital/Cancer Hospital Affiliated to Inner Mongolia Medical University, Hohhot, China; ^4^ Department of Nuclear Medicine, Peking University Cancer Hospital Inner Mongolia Hospital/Cancer Hospital Affiliated to Inner Mongolia Medical University, Hohhot, China

**Keywords:** chylopericardium, a case report, esophageal cancer, radical surgery, thoracic

## Abstract

**Background:**

Postoperative chylpericardium is a rare clinical disease that often manifests as chest tightness, shortness of breathdyspnea, and other symptoms of pericardial tamponade. The etiological spectrum of chylopericardium is complex, but the disease is mainly idiopathic. Chylopericardium caused by thoracic surgery is rarely reported, both at home and abroad.

**Case summary:**

We report a case of a 65-year-old male patient who developed chylopericardium after thoracoabdominal combined incision and partial esophagogastric anastomosis plus lymph node dissection for 1 month. After pericardiocentesis and drainage, low-fat enteral nutrition, and parenteral nutrition, the patient was cured. Based on this case, this article reviews the literature on the diagnosis and treatment of chylopericardium after thoracic surgery.

**Conclusion:**

In conclusion, thoracic surgery (excluding cardiac surgery) can cause delayed chylopericardium. This condition is rarely reported in China, and only a few cases have been reported abroad. Thus, the diagnosis is likely to be missed or misdiagnosed. Early diagnosis and treatment are important to reduce patient discomfort as much as possible.

## Introduction

Postoperative chylpericardium is caused by the accumulation of triglyceride-rich chyle in the pericardial cavity. Any disease that causes compression, obstruction, or rupture of the thoracic duct and its branches may cause chyle to enter the pericardium. Therefore, the etiological spectrum of this disease is complex. At present, there are many reports of idiopathic chylopericardium. Occasionally, chylopericardium can occur after cardiac surgery. Other rare causes of chylopericardium include venous thrombosis of the upper extremity and tumors (1–3). However, chylopericardium is rare.

Current medical treatment for chylopericardium can achieve rapid symptom improvement, and includes pericardiocentesis and drainage plus a high-protein/low-fat diet or parenteral nutrition. However, the disease is prone to recurrence. Surgical treatment can achieve a better long-term prognosis, and common surgical methods include thoracic duct ligation and pericardial fenestration (4).

In this study, we report a case of a 65-year-old man who developed delayed chylopericardium 1 month after radical resection of esophagogastric junction tumor. He underwent pericardiocentesis and drainage, a high-protein/low-fat diet, and upper extremity intravenous thrombolysis and anticoagulation. The patient’s symptoms improved, he did not require surgical intervention, and there was no recurrence during follow-up.

## Case presentation

### Chief complaints

The patient, who was a 65-year-old male, was admitted to the hospital on 27^th^ July 2021, mainly due to intermittent upper abdominal discomfort with nausea and vomiting for more than 10 days. After admission, gastroscopy showed an irregular bulge 38–41 cm from the incisors, which bled easily when touched. Pathological biopsy results showed poorly differentiated cancer. Enhanced chest computed tomography (CT) showed cardia and gastric fundus wall thickening, which was considered as Ca. There were multiple enlarged lymph nodes in the left cardia, lesser curvature, greater gastric curvature, and hepatic hilum. The upper gastrointestinal angiography findings were consistent with the gastric fundus body area of the cardia (carcinoembryonic antigen 72-4 concentration: 18.4 U/mL). The rest of the laboratory examinations showed no abnormalities. The examination did not reveal any contraindications to surgery. Thus, on 4^th^ August 2021, partial esophagectomy and total gastrectomy with the jejunum, followed by jejunal anastomosis, The stomach was separated from the esophagus to the lower pulmonary vein level, and the perigastric vessels and mesentery were cut off. The esophagus and whole stomach were cut off, and the jejunum and proximal esophagus were manually anastomozed end-to-side (type B-II). as well as lymph node dissection were performed. The left cardia, right cardia, lesser curvature, greater gastric curvature, hepatic hilum, and lymph nodes adjacent to the inferior pulmonary ligament and subcarina were successfully operated. The postoperative pathological report showed hepatoid adenocarcinoma of the esophagogastric junction (pT4N3M0, stage IV). The patient recovered well and was discharged from hospital. Three weeks after surgery, the patient was admitted to the hospital because of chest tightness and fatigue, and he could not lie in a supine position.

### History of past illness

The patient had a 2-year history of cerebral infarction, which was managed with long-term aspirin; a 7-year history of hypertension with a blood pressure of up to 160/90 mmHg, which was managed with regular oral nifedipine sustained-release tablets; and a 5-year history of type 2 diabetes mellitus, which was managed with oral repaglinide for blood glucose control.

### Personal and family history

The children and wife are healthy and have no history of disease. Parents and siblings have no history of disease.

### Physical examination

A physical examination revealed that the patient was clammy with a cold body, orthopnea, a blood pressure of 70/50 mmHg, a heart rate of 170 beats/min, expansion of the cardiac borders on both sides, distant heart sounds, bilateral lung breathing Low voice, dullness on percussion of bilateral lungs, and palpable pitting edema in the lower extremities.

### Laboratory examinations

The pericardiocentesis tube drained 1500 mL of milky white fluid intermittently, Laboratory examinations showed that the triglyceride concentration was 637 mg/dL, and the protein content was 43.2 g/Lin the Pericardial effusion. The right thoracic drainage tube drained 1200 mL of pale yellow pleural fluid,The Rivalta test of pleural effusion was negative, and it was considered to be leakage.

### Imaging examinations

**Figure 1:**
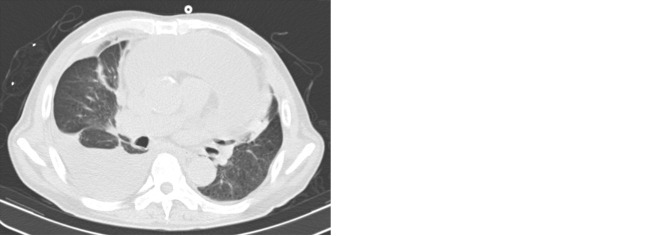
Chest CT mediastinal window and lung window of the patient 1 month after surgery.

Urgent chest CT showed bilateral pleural effusion and massive pericardial effusion, as well as insufficiency of the middle and lower right lung lobes (**Figure 1**). Echocardiography showed a large amount of pericardial effusion. Right thoracentesis and pericardiocentesis were urgently performed. Systemic vascular ultrasound showed right internal jugular vein and subclavian vein thrombosis (partial type), right brachial vein thrombosis (complete type), bilateral lower-extremity intermuscular vein thrombosis (complete type), and right small saphenous vein thrombosis (complete type).

### Initial diagnosis

The patient was diagnosed with 1) chylopericardium, 2) bilateral pleural effusion, 3) venous thrombosis of the lower extremities, 4) right brachial vein, internal jugular vein, and subclavian vein thrombosis, and 5) esophagogastric junction hepatoid adenocarcinoma (pT4N3M0, stage IV).

### Treatment

Considering the patient’s diagnosis of chylopericardium, a high-protein/low-fat enteral diet and partial parenteral nutrition were administered. After consultation with the interventional department, an inferior vena cava filter was implanted, and systemic thrombolysis and anticoagulation were performed. One week later, the patient’s symptoms (chest tightness and dyspnea) improved, and the pericardial drainage volume was 5 mL/day (pale yellow in color). After 24 hours of clipping, the patient had no discomfort. Echocardiography showed no obvious effusion in the pericardium or thoracic cavity, so the patient was extubated and discharged.

### Outcome and follow-up

According to the pathological stage, postoperative adjuvant therapy should have been carried out, but considering the weak physical condition, the patient refused chemotherapy, but the patient reported taking oral Chinese medicine after surgery.There was no recurrence of chylopericardium in the three-month and six-month follow-up of the patient, and the patient’s tumor showed no signs of recurrence.

## Discussion

Chylopericardium is mainly caused by the accumulation of triglyceride-rich chyle in the pericardial cavity, which was first reported by Hasebrock in 1888. The incidence of this disease is extremely low. Idiopathic chylopericardium is more common than chylopericardium after surgery. Dib et al. (2) analyzed 33 patients with chylopericardium. hairy. The symptoms of chylopericardium vary in severity and are related to the speed and amount of effusion. If effusion is severe, symptoms of pericardial tamponade, such as chest tightness, dyspnea, and an inability to breathe in a supine position, may be experienced by the patient. The patient in the present case developed the above symptoms 3 weeks after surgery, and his symptoms gradually worsened. At the time of consultation, the patient’s heart rate was fast, his blood pressure was low, heart sounds were distant, and his whole body was clammy and cold, illustrating insufficient perfusion. Due to the high pressure in the pericardial cavity, venous return to the heart decreased and bilateral leakage occurred.

The diagnosis of chylopericardium mainly depends on pericardiocentesis, which removes fluid that is milky white in appearance. Sometimes, a small amount of bleeding is caused by puncture, which appears pink. A specific gravity of 1.014-1.025, a low cholesterol concentration and a high triglyceride concentration (triglyceride concentration of >500 mg/dL; cholesterol/triglyceride ratio of <1), a high protein content (>35 g/L), abundant lymphocytes, adding which quickly become clear after the addition of ether. After Sudan III staining, fat globules can be seen under the microscope (5, 6). The patient in the present case underwent chest CT and echocardiography, which showed a large amount of pericardial effusion, and the pericardial drainage fluid was milky white in color. Laboratory examinations showed that the triglyceride concentration was 637 mg/dL, and the protein content was 43.2 g/L. liquid. The results are consistent with those of previous studies. In addition, Hemrom et al. (7) proposed that radionuclides can be used to diagnose chylopericardium and to identify the site of lymphatic damage, but due to the poor basic condition of the patient in the present case, radionuclide examination was not performed on admission.

There are many causes of chylopericardium, and some studies have shown that more than half of them are primary (2). Riquet et al. (8) analyzed pericardial lymphatic drainage in 90 autopsy cases. They concluded that primary chylopericardium may be congenitally associated with damage to the thoracic duct and its branch valves communicating with the pericardial lymphatic vessels, as well as abnormal communication between the lymphatic vessels and the pericardial cavity. Moreover, cardiac surgery is a common cause of chylopericardium (9% of cases), second only to idiopathic chylopericardium (2). Kan et al. (9) analyzed the causes of chylopericardium in 29 patients after cardiac surgery by reviewing the literature. The authors found that 10 patients had chromosomal abnormalities (trisomy 21 syndrome in all cases), which increases lymphatic vessel permeability. In addition, in 26 patients, the aorta and pulmonary artery were dissected near the right lymphatic duct, which may be related to the above factors. Other reports have suggested that chylopericardium can occur due to lymphatic damage into the thymus or pericardium, or to indirect damage during surgery (10, 11). In addition, some studies have shown that superior vena cava or subclavian vein thrombosis can obstruct lymphatic fluid return in the thoracic duct. When the pressure exceeds 15 cm H_2_O, chylopericardium can occur (12). Other causes of chylopericardium include malignant tumor and congenital lymphatic malformation, amongst others. We analyzed the possible etiology of late-onset chylopericardial effusion in the present case. Our findings were as follows. First, after radical resection of esophageal cancer, lymph node dissection in the abdominal and thoracic cavities, and esophagectomy, the jejunum replaced the esophagus to occupy part of the thoracic cavity volume, which may have oppressed pericardial lymphatic drainage. In addition, after total gastrectomy, the small intestine absorbs nutrients rapidly after eating, and the lymphatic fluid reflux load increases per unit time, which eventually leads to increased pericardial lymphatic pressure and rupture, which may have caused chylopericardium in the present case. Second, after admission, the patient was found to have thrombosis in the right internal jugular and subclavian veins. The thoracic duct mainly drains into the left subclavian vein, and the venous system of the left upper extremity of the patient was not abnormal. However, lymphatic drainage also occurs into the right subclavian vein, and we believe that subclavian vein thrombosis may have been a secondary cause of chylopericardium in the present case.

Most scholars believe that the first-line treatment for chylopericardium should be pericardial drainage alongside a high-protein/low-fat diet plus parenteral nutrition (4). Karaca et al. (13) proposed that somatostatin is effective in the treatment of chylopericardium caused by ascending aorta replacement, and effusion is also effective. Moreover, Fakhri et al. (14) reported successful treatment with steroids after pulmonary venous drainage in a patient with chylopericardium. Therefore, in the present case, we first performed pericardiocentesis and drainage. We simultaneously administered a low-fat diet and parenteral nutrition, and the amount of pericardial effusion gradually decreased. At this time, chest tightness and dyspnea were significantly relieved, and the patient was able sleep in the supine position.

Previous studies have shown that thrombosis in the jugular vein, subclavian vein, and superior vena cava can affect the lymphatic return of the thoracic duct, causing chylopericardium. According to a previous study, chylopericardial effusion disappeared after upper-extremity venous stenting or superior vena cava stenting, or after administration of thrombolytic anticoagulation (12). Thus, we cannot exclude venous thrombosis of the upper extremity as the cause of chylopericardium in the present case. After consultation with the interventional department, the patient underwent local thrombolysis and anticoagulation for symptomatic treatment. Pericardial effusion stopped after 1 week, and the patient’s symptoms improved. Thus, he was discharged from the hospital.

Some scholars believe that refractory chylopericardium can be treated with surgical intervention when pericardial drainage or conservative treatment fails. Among them, lower thoracic duct (near to the diaphragm) ligation/resection and pericardial fenestration are the most commonly used (15, 16). Some researchers also believe that simple ligation of the thoracic duct can achieve good results. In addition, pericardial–peritoneal shunt surgery and thoracic duct–internal jugular vein anastomosis lymphatic circuit reconstruction have demonstrated good short-term effects (17). In the present case, no obvious pericardial effusion was observed during follow-up, but the possibility of recurrence cannot be ruled out. If recurrence occurs later, surgical ligation of the thoracic duct may be considered.

## Conclusion

In conclusion, thoracic surgery (excluding cardiac surgery) can cause delayed chylopericardium. This condition is rarely reported in China, and only a few cases have been reported abroad. Thus, the diagnosis is likely to be missed or misdiagnosed. Early diagnosis and treatment are important to reduce patient discomfort as much as possible.

## Data availability statement

The original contributions presented in the study are included in the article/supplementary material. Further inquiries can be directed to the corresponding author.

## Ethics statement

Written informed consent was obtained from the individual(s) for the publication of any potentially identifiable images or data included in this article.

## Author contributions

PY and RH contributed to study design. BZ and YW contributed to drafting of the article. PY, BZ, and RH contributed to data collection. BH, RH and PY participated in data analysis and interpretation and led the article’s revision. All authors contributed to the article and approved the submitted version.
